# Nest desertion as an anti-parasitism strategy in hosts selects for late egg-laying behavior in cuckoos

**DOI:** 10.1016/j.isci.2023.108156

**Published:** 2023-10-06

**Authors:** Guo Zhong, Guixia Wan, Yuhan Zhang, Huahua Zhao, Longwu Wang, Wei Liang

**Affiliations:** 1 Ministry of Education Key Laboratory for Ecology of Tropical Islands, College of Life Sciences, Hainan Normal University, Haikou 571158, China; 2 School of Life Sciences, Guizhou Normal University, Guiyang 550001, China

**Keywords:** Biological sciences, Ornithology, Evolutionary biology

## Abstract

Studies have shown that brood parasites lay their eggs early in the egg-laying sequence of their hosts, providing them with the advantage of earlier hatching. However, common cuckoos (*Cuculus canorus*) appear to parasitize the nests of gray bushchat (*Saxicola ferreus*) during the late egg-laying stage. The bushchat often abandons parasitized nests in the early stages, but not in the late egg-laying stages, thus favoring late egg-laying by cuckoos. In this study, four experiments were conducted to determine whether gray bushchats employ a nest desertion strategy targeted at cuckoo parasitism. The results showed that nest desertion was significantly correlated with parasitism cues and occurred mainly during the hosts’ early egg-laying stage. Our study provides the first experimental evidence that nest desertion is an anti-parasitic strategy used by hosts in response to cuckoos. Additionally, our experiments demonstrated that the nest desertion is influenced by the trade-offs of investments in different egg-laying stages.

## Highlights

• Nest desertion by the host is an anti-parasitic strategy against cuckoo parasitism
• Nest desertion is influenced by the trade-offs of host investments for their nests
• Hosts desert their nests in early egg-laying stage in response to cuckoos

## Graphical abstract

**Figure:**
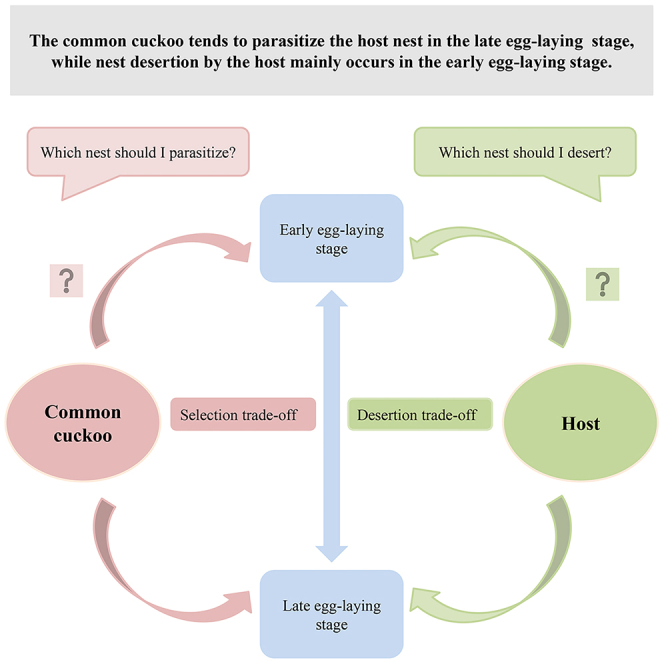

## Introduction

Brood parasitism is a widespread reproductive strategy throughout the animal kingdom, including birds, frogs, fish, and insects.^1^^,^^2^^,^^3^ The antagonistic interaction between avian brood parasites and their hosts has emerged as a model system for studying the “arms race” theory of coevolution.^4^^,^^5^ According to the natural selection theory, hosts have evolved various anti-parasitic adaptations in response to the high costs of parasitism.^1^^,^^6^ Host responses to parasitized nests are generally categorized into two types: acceptance and rejection (ejection of the parasite egg and nestling, burying of the parasite egg, and nest desertion).^7^^,^^8^ The abandonment of parasitized nests (eggs or nestlings) is an important anti-parasitic behavior.^9^^,^^10^^,^^11^^,^^12^ However, the mechanisms underlying nest desertion remain poorly understood.

The fitness costs associated with host reproduction due to brood parasitism facilitate the evolution of anti-parasitism adaptations among hosts.^6^ For example, most hosts exploited by cuckoos (*Cuculus* spp.) are small passerine birds that have evolved the ability to recognize and reject parasite eggs, owing to the high cost of parasitism.^1^ However, ejecting highly mimetic and large parasite eggs from some cuckoo hosts is difficult.^1^^,^^6^ Therefore, in such instances, nest desertion strategies are vital for cuckoo hosts, as nest desertion can lead to favorable reproductive adaptations.^7^^,^^13^^,^^14^ Various cues can elicit a reaction to parasitism, such as encounters with parasites at the nest, detection of parasite movements around the nest,^9^^,^^15^^,^^16^^,^^17^ and differences between parasite and host eggs.^18^^,^^19^^,^^20^^,^^21^ Observations of naturally parasitized nests have indicated that most hosts exhibit various degrees of desertion of parasitized nests.^14^ A study on the common redstart (*Phoenicurus phoenicurus*) demonstrated that the desertion rate of parasitized nests was significantly higher than that of non-parasitized nests.^11^ Therefore, comprehensive studies on anti-parasitism strategies are needed to elucidate the reasons and adaptive mechanisms underlying nest desertion in cuckoo hosts following parasitic encounters.

Consequently, several studies have explored the causes of nest desertion adaptation in hosts. First, nest desertion by parents is associated with a decrease in the number of eggs in the nest. For example, studies on cowbird hosts have found that the nest desertion rate of parents is positively correlated with a reduction in the number of eggs in the nests.^22^ This could be related to the reproductive trade-offs associated with parasitized nests.^23^^,^^24^ The choice to desert is an important adaptation because it becomes unproductive to invest further once the number of eggs falls below a certain reproductive threshold.^25^^,^^26^^,^^27^ Secondly, host encounters with parasites near the nest are important cues that lead to nest desertion.^7^^,^^28^^,^^29^^,^^30^ Studies on the reed warbler (*Acrocephalus scirpaceus*) have shown that when hosts spot a cuckoo dummy, they abandon their nests.^9^ Similar observations have been reported in cowbird hosts.^30^^,^^31^ However, other studies have found that encounters with parasites do not increase nest abandonment.^32^^,^^33^ Third, host nest desertion is triggered by non-mimetic parasite eggs. The behavioral responses of hosts to parasite eggs are influenced by egg characteristics and host body size.^34^^,^^35^^,^^36^ For example, Western Bonelli warblers (*Phylloscopus bonelli*) and marsh warblers (*Acrocephalus palustris*) ejected smaller model eggs but abandoned nests with large model eggs.^37^^,^^38^ However, despite the ejection of parasite eggs, some hosts still abandon their nests at times.^39^^,^^40^ In some cases, the presence of parasite eggs does not lead to nest desertion by the host.^30^

Finally, the perceived risk of nest predation may lead to nest desertion by hosts.^41^^,^^42^^,^^43^^,^^44^ For example, studies on gray fantails (*Rhipidura albiscapa*) found that stimulation by predators significantly increased nest desertion in hosts compared to controls without predators.^45^ Parasites may also prey on host eggs or chicks,^1^^,^^46^^,^^47^^,^^48^ but studies on nest defense have demonstrated that parasites such as common cuckoos are recognized more often by hosts as cues of parasitism than predation.^1^^,^^49^^,^^50^^,^^51^^,^^52^ Field observations suggest that the nest desertion behavior of hosts is primarily triggered by cues of parasitism, suggesting that nest desertion is an important adaptation mechanism of anti-parasitism behavior.^11^^,^^14^^,^^53^^,^^54^ While previous studies have investigated host adaptation to nest desertion from different angles, they have rarely examined the impact of parental investment trade-offs on nest desertion at different stages of egg-laying.

**Figure 1:**
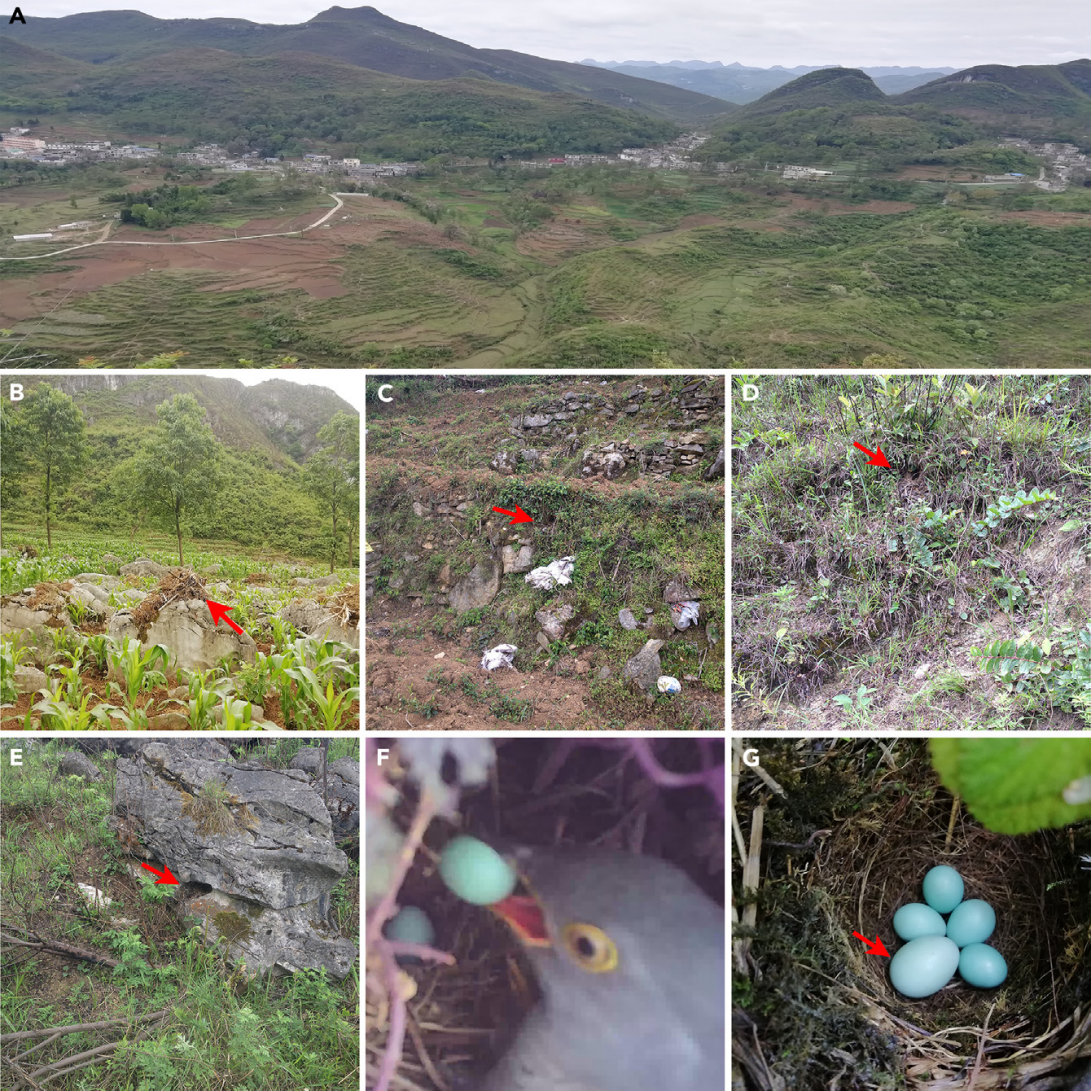
The host's nesting habitat and the parasitic process of the cuckoo Landscape of the study site (A), various breeding habitats and nest-sites of the gray bushchat (nesting on cultivated land (B), barren slopes, and near the grassroots (C and D) or rock cover (E) in scrub forests), the egg pecking behavior of the common cuckoo (F), and the eggs of the cuckoo and gray bushchat host (G). The arrow is pointing at the location of the nest and cuckoo egg.

Gray bushchat (*Saxicola ferreus*) is the dominant host of the common cuckoo (*Cuculus canorus*) (Table 1, Figure 1).^55^ Our observations over five breeding seasons revealed that parasitism of the gray bushchat by the common cuckoo tended to occur during the late egg-laying or incubation period, whereas nests parasitized during the early egg-laying stages of the host were more likely to be abandoned (see the Results section). This phenomenon is in stark contrast to other cuckoo hosts, such as the Oriental reed warbler and the great reed warbler, where the cuckoo prefers to lay its eggs in nests that are early in the laying sequence.^56^^,^^57^^,^^58^ To date, there have been few studies on the causes and patterns of host nest desertion in parasitic brood systems.^30^^,^^31^^,^^38^^,^^59^

**Table 1:** Parameters for parasite eggs (natural and experimental parasitism) and host eggs (mean = x (SD = y))

Types of egg	Egg mass (g)	Egg length (mm)	Egg width (mm)	Egg volume (cm^3^)
Parasite egg (n = 15)	3.3 (0.3)	22.1 (0.5)	16.8 (0.4)	3.2 (0.2)
Host egg (n = 21)	2.1 (0.1)	18.2 (0.7)	14.5 (0.5)	2.0 (0.3)
Model egg (n = 20)	3.6 (0.0)	21.1 (0.1)	16.5 (0.0)	3.0 (0.0)
Budgerigar egg (n = 18)	1.9 (0.2)	18.6 (0.6)	15.2 (0.5)	2.2 (0.2)

This study aimed to experimentally demonstrate whether nest desertion adaptation of the gray bushchat host is related to cues of parasitism and to explore nest desertion patterns. We used natural cuckoo parasitism and host nest desertion data from five breeding seasons to calculate the distribution of egg-laying timing and host nest desertion patterns. Second, we determined whether nest desertion patterns were correlated with parasitic events by designing experiments on parasitism with exposure to different cues (see the field experiments section). Based on this, our first conclusion was that if the nest desertion adaptation of the experimental groups was similar to that of natural parasitism (nest desertion behavior occurs mainly in the early laying stages rather than in the later stages), it would suggest that the nest desertion pattern of gray bushchat is related to an investment trade-off by host parents. The second conclusion was that the different rates of nest desertion between parasitized and non-parasitized nests suggest that nest desertion is an anti-parasitic adaptation that is triggered by cues of parasitism. Furthermore, if the ejection rates of Group 1 (white model eggs) and Group 4 (white budgerigar eggs) differed, but the nest desertion rate remained the same, we inferred that the nest desertion adaptation of the gray bushchat may not be related to the cost of ejection.

## Results

During the five breeding seasons (March–August) of 2018–2022, 1,116 gray bushchat nests were found, 8.2% of which were parasitized by the common cuckoo. In cases where the number of eggs in the parasitized clutch was determined, we found a significantly higher proportion of parasitism in the late egg-laying (4–5 eggs) than in the early egg-laying stage (1–2 eggs) (16 vs. 50; chi-squared test for given probabilities, χ^2^ = 17.515, degree of freedom [*df*] = 1, p = 0.001). Nests parasitized in the early egg-laying stages were more likely to be abandoned by the host than nests in the late egg-laying stages (Bonferroni confidence intervals: 0.099 ≤ P_1_ ≤ 0.451 [early stage] vs. 0.014 ≤ P_2_ ≤ 0.3 [late stage]; n = 66; Table 2).

**Table 2:** Hosts behavioral responses (acceptance, ejection, and nest abandonment) to parasite eggs of manipulation and natural parasitism

Type of parasitism	Natural parasitism		Group 1		Group 2		Group 3		Group 4		Group 5	
	1–2 eggs	4–5 eggs	1–2 eggs	4–5 eggs	1–2 eggs	4–5 eggs	1–2 eggs	4–5 eggs	1–2 eggs	4–5 eggs	1–2 eggs	4–5 eggs
Ejection	0	0	5	0	1	2	0	0	16	20	0	0
Nest desertion	14	8	8	0	4	1	8	0	8	0	1	0
Acceptance	2	42	15	33	24	25	20	29	8	11	12	13
Sample (N)	16	50	28	33	29	28	28	29	31	31	13	13

In the early stage of Group 4, there was one sample of the host rejecting budgerigar eggs before abandoning the nest.

**Figure 2:**
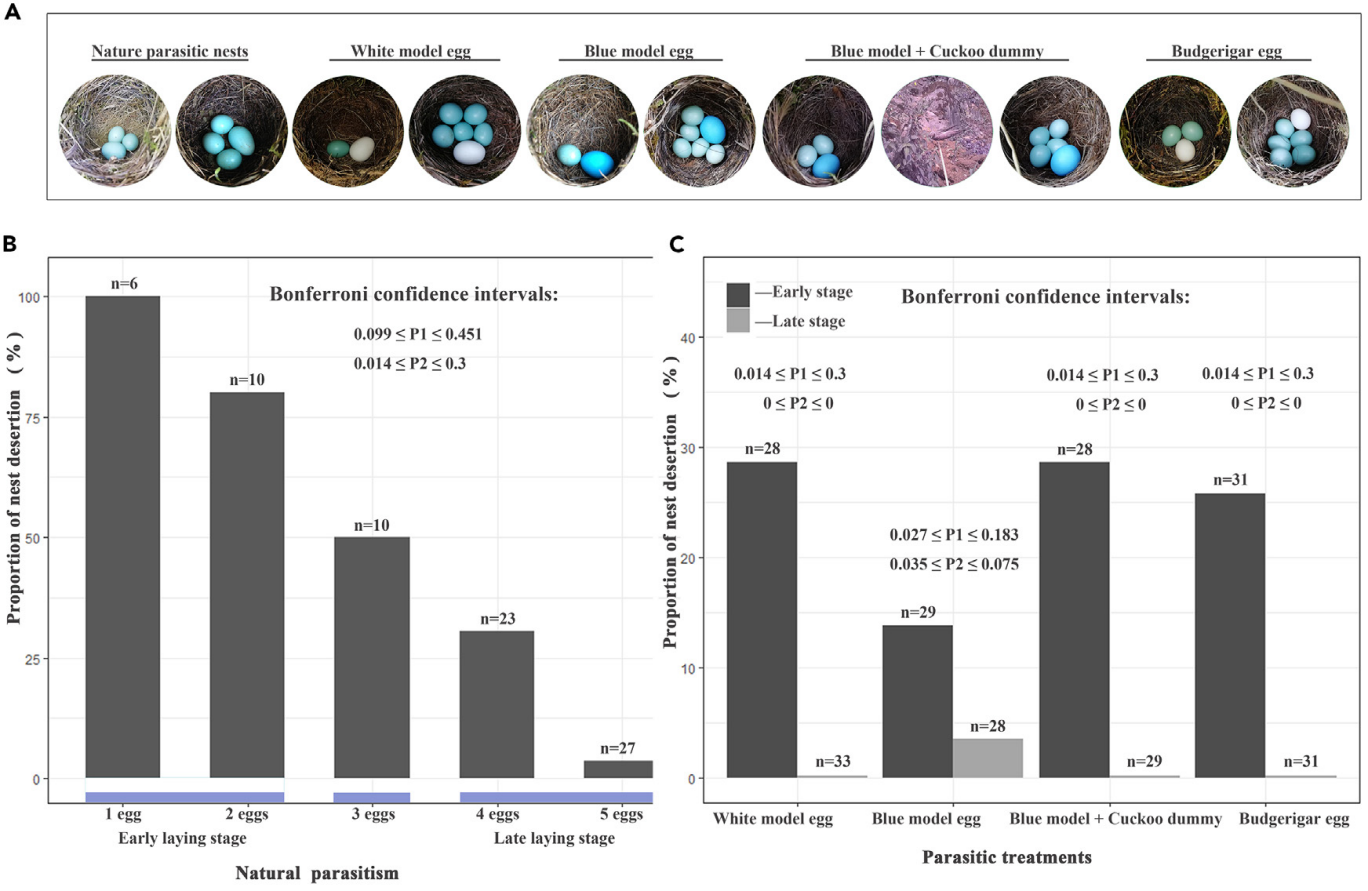
Comparison of host nest desertion rates of natural cuckoo-parasitized nests and nests subjected to experimental parasitism at different egg-laying stages (A) Schematic diagram of the experiment. (B) Distribution of natural parasitic nests and nest desertion rate. (C) Desertion rate of experimental parasitic nests at different egg-laying stages. In the second chart, Groups 1–4 represent manipulation parasitic experiments. Model eggs are similar in size to true cuckoo eggs, and Budgerigar eggs are similar in size to host eggs. In the charts, “Early stage” replaces the early egg-laying stage, and “Late stage” replaces the late egg-laying stage. P1 represents the Bonferroni confidence interval for the early egg-laying stage; P2 represents the Bonferroni confidence interval for the late egg-laying stage.

We compared the four groups of the parasitic manipulation experiments and found that host adaptation of nest desertion was selected in the early rather than late egg-laying stages (Figure 2). A comparison of the four manipulation experiments in the early egg-laying stage revealed no significant differences among the four groups (χ^2^ = 0.778, *df* = 3, p = 0.378). Furthermore, there was a significant difference in nest desertion rates between the three groups of non-parasitized (control) and parasitized nests (natural and experimental parasites) during the early egg-laying stage (Fisher’s exact test, p < 0.001). We conducted post-hoc comparisons between the treatments and found that all p values were <0.001 (Table 3).

**Table 3:** Comparison of host desertion rates of different parasitic manipulation experiments in the same stage (early or late egg-laying)

Dependent variable	Type of parasitism cue	χ^2^	*df*	*p*
Early egg-laying	Group 1–4	0.778	3	0.378
	Group 1 vs. Group 2	1.872	1	0.171
	Group 1 vs. Group 4	0.033	1	0.857
	Group 3 vs. Group 5		1	0.228
	Control vs. Group 1–4	8.390	1	0.004
	Control vs. Nat parasitism	35.933	1	0.000
	Group 1–4 vs. Nat parasitism	26.027	1	0.000
Late egg-laying	Group 1–4 vs. Nat parasitism		1	0.000

In all-natural and experimental parasitic experiments, egg ejection occurred mainly in group 4 (Table 2). The comparative study found that even though there was a significant difference in ejection rates between Group 1 and Group 4 (early stage: χ^2^ = 19.829, *df* = 1, p < 0.001; late stage: χ^2^ = 19.49, *df* = 1, p < 0.001), there was no difference in nest desertion patterns (χ^2^ = 0.033, *df* = 1, p = 0.857), and nest desertion only occurred in the early egg-laying stages (Table 2). In Group 5, only one nest was abandoned during the early egg-laying stage, and there was no significant difference in the rate of nest desertion in Group 3 (Fisher’s exact test, p = 0.228) (Table 3).

## Discussion

Our study demonstrated that the desertion of naturally parasitized nests by gray bushchat hosts was triggered by cues of parasitism from the common cuckoo rather than by disturbances associated with nest visits during the experiment. Additionally, the pattern of parasitized nest desertion by gray bushchats depends on the trade-off of investment at different egg-laying stages, independent of the host’s ejection costs of anti-parasitism. Our study demonstrated that nest desertion is an important anti-parasitism strategy for gray bushchats in response to common cuckoo parasitism, and experimental verification showed that this anti-parasitism adaptation exists mainly in the early egg-laying stage. In the late egg-laying or incubation stage, the gray bushchat rarely abandons its nests, despite the presence of cuckoo parasitism.

In this study, a similar pattern of nest desertion by gray bushchats was elicited in response to both experimentally and naturally parasitized nests, occurring during the early rather than late egg-laying stages. Furthermore, no nest desertion due to nest visits occurred in the non-parasitized nest control group, suggesting that the desertion of parasitized nests by the gray bushchat host is indeed an anti-parasitism strategy, as these responses were elicited by parasitism cues. Similar studies based on dummy stimuli demonstrated that encounters with parasites cause nest desertion.^9^^,^^30^ Some studies have suggested that host nest desertion behavior is related to the ejection costs of parasite eggs.^34^^,^^35^^,^^60^ However, our results do not support the ejection cost hypothesis, as we found that in the late egg-laying period, gray bushchats rarely abandoned larger simulated or non-mimetic cuckoo eggs, whereas budgerigar eggs similar in size to host eggs were abandoned in the early egg-laying stage. Studies have shown that some hosts choose to abandon their nests instead of rejecting the parasite eggs.^39^^,^^40^ In addition, we found that only 16% (n = 50) of the cuckoo eggs were abandoned during the late egg-laying or incubation period, which could be mainly due to excessive parasitic disturbances caused by cuckoos. In four cases, we observed at least six attempts at parasitism by cuckoos before success, suggesting that nest desertion was not due to host ejection.^61^

Notably, the nest desertion behavior of gray bushchats exhibited adaptations for the egg-laying stage. We propose that parental investment at different egg-laying stages influences the host’s nest desertion decisions. According to the parental investment theory, parents are more likely to abandon breeding nests with lower offspring values than those with higher offspring values.^24^^,^^25^^,^^26^^,^^27^^,^^62^ Similar studies have found that the desertion rate of nests parasitized by chipping sparrows gradually decreases in the late egg-laying stage,^30^ and a significantly higher desertion rate was found before egg laying than after egg laying.^63^ However, studies on natural parasitism in yellow warblers, the hosts of cowbirds, have revealed their predisposition to bury individual eggs in the nest and abandon their nests after three eggs have been laid.^64^ Yellow warbler nest desertion behavior might be attributed to factors beyond the parasitic response.^14^^,^^64^ Previous studies have speculated that the cost of egg ejection affects host desertion behavior, suggesting that hosts of highly mimetic parasite eggs are more inclined toward nest desertion; however, this was not supported by comparative studies,^61^ possibly because these studies did not consider the influence of reproductive cost trade-offs on host nest desertion behavior. In contrast, our experimental data showed that among the large non-mimetic model eggs, those in the early laying stage were abandoned at a comparable or slightly higher rate than simulated cuckoo eggs. However, in the late egg-laying stage, nest desertion behavior was rarely observed, regardless of the degree of parasite egg mimicry or the possibility of egg ejection. Additionally, our results showed that the degree of simulation and size of parasite eggs influenced host ejection and not nest desertion behavior, suggesting that the nest desertion strategy of gray bushchats is influenced by the degree of parasite egg mimicry in the pre-egg-laying stage, as it determines whether the host will eventually abandon the nest. However, in the late egg-laying stage, the effects of the degree of mimicry became less important as the gray bushchat does not abandon the nest owing to ejection costs, which explains why cuckoo hosts often accept parasite eggs despite recognizing them.^1^^,^^6^

The desertion rate of all experimentally parasitized nests was significantly lower than that of naturally parasitized nests during the early egg-laying stage, particularly in Group 2, which could be attributed to the different parasite encounters during the two processes,^7^^,^^11^^,^^28^^,^^65^ as natural parasitism entails diverse cues, and the levels of exposure to these cues are different from those experienced during experimental conditions. For example, multiparasitised nests are more likely to be abandoned,^66^ which could be related to multiple nest visits by cuckoos. We also observed that nests with four to five eggs were parasitized only after frequent parasitism attempts and host attacks, eventually leading to nest desertion. However, the reason behind the significantly lower desertion rate of experimentally parasitized nests compared to that of naturally parasitized nests remains unknown. Although our data suggest that hosts use nest desertion as an anti-parasitism strategy stimulated by cues of parasitism and that this pattern of nest desertion correlates with the investment trade-off of parents at different egg-laying stages, caution should still be taken when concluding that nest desertion serves as a host response to parasitism. Nest desertion is not always the only host response to parasitism^22^ because parasites often act as predators too.^67^^,^^68^^,^^69^ Future research should further clarify how cuckoo hosts, such as gray bushchats, identify parasitism cues in the context of the complex roles of both nest parasites and predators.

In conclusion, our study demonstrated that nest desertion is an important anti-parasitic strategy for this cuckoo host. The choice of this adaptation strategy depends on the parents’ investment trade-offs at different egg-laying stages of the host, implying that the extent to which parasite eggs are stimulated in the early rather than the late egg-laying stage is important for nest desertion adaptation by the host. Furthermore, the synchronized egg-laying time exhibited by the common cuckoo in this parasitic system might also suggest coevolution with host nest desertion, favoring late egg-laying behavior in cuckoos; however, this requires further verification. To the best of our knowledge, this is the first study to demonstrate that the host’s anti-parasitism strategy of nest desertion is related to the investment trade-off of parents at different egg-laying stages. However, the prevalence of this nest desertion pattern in gray bushchat hosts requires further validation by investigating more avian brood parasitism systems and host species.

### Limitations of the study

Our study provides the first experimental evidence that nest desertion is an anti-parasitic strategy used by gray bushchats in response to cuckoo parasitism. However, whether the host’s pattern of nest abandonment in this study in relation to investment trade-offs varies differently in other parasitic systems needs to be further verified across different geographic populations or species. In addition, future work should also investigate whether the synchronized egg-laying time exhibited by the common cuckoo in this host-parasite system may favor late egg-laying behavior in cuckoos.

## STAR★Methods

### Key resources table

**Table:** 

REAGENT or RESOURCE	SOURCE	IDENTIFIER
**Deposited data**		

all videos and extracted data	This paper	https://doi.org/10.5061/dryad.866t1g1vs

**Software and algorithms**		

Leawo video Converter Software	This paper	http://www.downza.cn/soft/12064.html
R Software	This paper	https://www.R-project.org/.

### Resource availability

#### Lead contact

Further information and requests for resources and reagents should be directed to and will be fulfilled by the lead contact, Wei Liang (liangwei@hainnu.edu.cn).

#### Materials availability

DOIs are listed in the key resources table.

### Experimental model and study participant details

This study was conducted during the 2018–2023 breeding seasons (March–August) in Liuzhi (26°13′ N, 105°42′ E) of Guizhou, Southwestern China. The study site is located in the northern temperate monsoon climate zone at an elevation of 1070–1657 m. The landscape of the study site includes villages, cultivated land, scrub forests, and barren slopes (Figure 1A).^70^

The grey bushchat is a small passerine bird that breeds at the study site from early April to late July, nesting on cultivated land (Figure 1B), barren slopes, and near grassroots (Figures 1C and 1D) or rock cover (Figure 1E) in scrub forests with relatively secluded nests. Females typically lay clutches of 4 to 5 eggs (rarely 3 to 6 eggs). They begin incubating the eggs when the clutch is full, or one day earlier. The males guard the nest during incubation. In addition, according to our observations, the host hardly ejected true cuckoo eggs but usually used an ejection method for experimental eggs similar in size to its own. In contrast, common cuckoos usually migrated to the study site in late April, with grey bushchats as their main hosts. They usually picked a host egg (Figure 1F) when laying, and the parasite eggs were highly similar to the host eggs in background color (Figure 1G, Table 1).

#### Ethical standards

The experiments complied with current laws in China. The experimental procedures were performed per the guidelines of the Animal Research Ethics Committee of the Hainan Provincial Education Centre for Ecology and Environment, Hainan Normal University (No. HNECEE-2012-004) and the Experimental Animal Ethics Committee of Guizhou Normal University (No. 2021001).

### Method details

#### Field experiments

The area was systematically searched for active nests during the breeding season each year, and breeding status was recorded (e.g., nesting, egg laying, egg incubation, and brood rearing). The data collected for each nest included the following: the date of the first egg, clutch size, and incubation period. The location of each nest was marked with a GPS kit, and photos of the nests and eggs were taken with a camera. Nests were then checked periodically (2–3 d) to record whether they had been parasitized by cuckoos and the number of eggs in the clutch at the time of parasitism. To reduce the probability of natural cuckoo eggs being abandoned in the experiment and the need for subsequent studies on cuckoo eggs (parasite eggs in the early laying stages are easily abandoned by the host), some of the newly parasitized eggs were replaced with cuckoo eggs that had been abandoned at the time of discovery. The cuckoo eggs were manually incubated and then placed in the original nest or a nest of a similar age after hatching, in cases where the original parasitized nest was abandoned or predated. To verify the nest desertion patterns of hosts in response to parasitized nests, we designed different simulated parasitism experiments by dividing the hosts into early and late egg-laying groups (1–2 eggs vs. 4–5 eggs) as follows:

In Group 1, as no egg recognition ability has been reported for grey bushchat, the host was first stimulated with non-mimetic white model eggs (both cuckoo and host eggs were pure blue, see Figure 1F) to test the host’s recognition ability and nest desertion behavior in response to non-mimetic eggs (i.e., one white model egg was placed directly into the host’s nest and checked once every 2–3 d). The experiment concluded after 6 d.^59^ (Soler et al. 2015). We recorded the host's behavioral response to the parasitized nest during the experimental cycle. These responses were: (1) Ejecting only the parasite egg, but not deserting the nest, (2) Ejecting both the parasite egg and deserting the ne st, (3) No-ejective but abandoning the nest; (4) No-ejective and not deserting nest. In Group 2, based on the color of the cuckoo eggs, we used mimetic model eggs (blue model eggs) to explore whether the host nest desertion response was related to the degree of egg mimicry, following the same procedure as that used for Group 1. In Group 3, the natural parasite egg-laying process was simulated by placing a cuckoo specimen at a distance of 0.5–1 m from the nest while introducing a model egg into the nest. This approach was inspired by studies suggesting that host nest desertion is related to encounters with parasites.^8^^,^^14^ The experiment was conducted from 6:30 AM to 6:30 PM. Host behaviors such as attacking the specimen (see ESM Videos S1 and S2), ignoring the specimen, or watching the nest for over 1 min were videotaped(Uniscom-T71, 70 × 26 × 12 mm; Mymahdi Technology Co. Ltd., China) and observed using binoculars. The nests were periodically checked. The host nest desertion response in the first three groups of experiments could be related to the cost of egg ejection, as the experimental eggs were model eggs that were similar in size and quality to real cuckoo eggs, but difficult to peck. Thus, an additional experimental group (Group 4) was included in which real white budgerigar (*Melopsittacus undulatus*) eggs (trade-permitted non-fertilized eggs), which are similar in size and quality to the host eggs, were placed directly into the host nests, and their status was periodically observed. In Group 5, Oriental Turtle-dove specimen was used as an innocuous sympatric control species to verify that nest desertion was triggered in response to the sight of the parasite near the nest. This control involved achieving the same frequency of nest-visiting activity for some early egg-laying (1–2 eggs) non-parasitized nests. This was done to determine whether host nest desertion was associated with a cue of parasitism or an artificial stimulus for nest visitation.

Video S1. Host parents attacked the cuckoo dummy near the nest

Video S2. Host parents attacked the dove dummy near the nest

### Quantification and statistical analysis

Pearson’s chi-square test was used to compare the distribution of naturally parasitized nests in the egg-laying stages (early and late egg-laying stages) of the host, where samples with indeterminate clutch size and those with three eggs (late egg-laying stage for individuals with a full clutch size of four eggs, and middle egg laying stage for those with a full clutch size of five eggs) were excluded from the analysis. Thereafter, Pearson’s chi-square test or Fisher’s exact test were used to test for differences in host desertion rates of different parasitic manipulation experiments at the same stage (early or late egg laying), excluding nests with uncertain clutch size or desertion status from the analysis. In addition, we calculated the Bonferroni confidence intervals of nest desertion for each stage(P1: Bonferroni confidence intervals for early egg-laying stage; P2: Bonferroni confidence intervals for late egg-laying stage) in natural and manipulative parasitism per the method described by Byers et al.^71^ All statistical analyses were performed using R (R_3_._5.2_)^72^ and 'RStudio',^73^ and all tests were two-tailed at a 0.05 significance level, with egg parameters expressed as mean = x (SD = y).

## Data Availability

All the datasets of tracks and videos are available at the Dryad digital repository, the DOI is listed in the key resources table. Any additional information required to reanalyze the data reported in this paper is available from the lead contact upon request.
